# Syntaphilin Regulates Epithelial-Mesenchymal Transition and Metastasis in Gastric Cancer via the FAK/NF-κB/MMP-9 Signaling Pathway

**DOI:** 10.3390/ijms27156564

**Published:** 2026-07-23

**Authors:** Hye Jin Choi, Jong Min Park

**Affiliations:** College of Oriental Medicine, Daejeon University, Daejeon 34520, Republic of Korea; haejinchoi@hanmail.net

**Keywords:** syntaphilin, gastric cancer, EMT, FAK, MMP-9

## Abstract

Syntaphilin (SNPH), initially considered a neuron-specific protein, has recently been found to be widely expressed across various cancers. Mechanistically, SNPH inhibits mitochondrial transport to the cortical cytoskeleton, thereby suppressing cancer cell migration and metastasis. Although SNPH is known to participate in the metastatic progression of multiple malignancies, its precise underlying mechanism remains obscure. In this study, we investigated the role of SNPH in the epithelial–mesenchymal transition (EMT) and invasiveness of gastric cancer cells. Knockdown of SNPH in SNU-638 gastric cancer cells significantly enhanced their migratory and invasive capacities by approximately 1.4-fold and 2.5-fold, respectively. This knockdown concurrently increased focal adhesion kinase (FAK) phosphorylation, upregulated the EMT marker vimentin, and increased the expression of key EMT-related transcription factors, including Snail, Slug, and Twist. Furthermore, SNPH depletion induced the phosphorylation of nuclear factor kappa B (NF-κB), a transcription factor regulating matrix metalloproteinase-9 (MMP-9), which subsequently upregulated MMP-9 mRNA expression. This cascade promotes extracellular matrix degradation, thereby increasing metastatic potential. Notably, these phenotypic and molecular changes were completely reversed upon SNPH overexpression in SNU-638 cells. Taken together, our results demonstrate that SNPH deficiency enhances the migration and metastatic potential of gastric cancer cells by driving EMT and invasion via the FAK/NF-κB/MMP-9 signaling pathway. Consequently, we propose that SNPH represents a novel biomarker and a promising therapeutic target for mitigating gastric cancer metastasis.

## 1. Introduction

Gastric cancer (GC) remains one of the most prevalent malignancies in South Korea [1,2]. However, pronounced tumor heterogeneity, a shortage of reliable biomarkers for patient stratification, and a paucity of effective therapeutic targets severely hinder accurate prognostic prediction and the development of novel interventions [1]. Despite recent advancements in surgical techniques and adjuvant therapies, the survival rate of patients with GC remains suboptimal due to frequent metastasis and disease recurrence [3]. Therefore, elucidating the precise molecular mechanisms driving GC metastasis is imperative to improve patient survival outcomes [4,5,6].

Epithelial–mesenchymal transition (EMT) is a dynamic cellular process wherein polarized, adherent epithelial cells transform into a migratory and invasive mesenchymal phenotype. Although EMT is essential for normal embryonic development and tissue wound healing, its pathological activation contributes significantly to fibrosis and cancer progression. By enhancing cancer cell motility, EMT drastically increases metastatic potential [7,8,9]. Consequently, the initiation of metastasis inherently relies on EMT-driven invasion. This complex transition is orchestrated by a multitude of distinct molecular events, including the activation of specific transcription factors, altered expression of cell surface proteins, reorganization of the cytoskeletal framework, production of extracellular matrix (ECM)-degrading enzymes, and dysregulation of specific microRNAs [10,11].

Syntaphilin (SNPH) is a mitochondrial docking protein that functions as a “static anchor.” While it has traditionally been recognized as a critical regulator of axonal mitochondrial transport in neurons [12], emerging evidence highlights its pivotal role in cancer metastasis [13,14]. Mechanistically, as a representative cytoskeletal anchor, SNPH increases the binding avidity between mitochondria and microtubules and inhibits mitochondrial ATPase activity, thereby impeding organelle motility [15,16]. In the context of oncology, recent studies suggest that the dysregulation of SNPH is closely linked to the epithelial–mesenchymal transition (EMT). For instance, the ubiquitination-mediated functional degradation of SNPH in cancer cells accelerates mitochondrial trafficking to the cortical cytoskeleton, which subsequently enhances tumor invasion and metastasis [12,17]. Furthermore, clinical evidence from a cohort of patients with localized and metastatic prostate cancer revealed spatial regulation of SNPH expression; specifically, low SNPH expression within the central tumor mass was strongly associated with metastatic disease [18]. Given that SNPH-mediated mitochondrial immobilization directly modulates cellular energy metabolism and motility—both of which are critical for supporting the high energetic demands of cell migration during EMT—SNPH likely serves as a key mediator regulating the EMT program in malignant cells [12,13]. Mechanistically, the spatial redistribution of mitochondria within cancer cells is closely linked to metastatic progression. Previous studies have demonstrated that trafficking of mitochondria toward the cell periphery or cortical cytoskeleton can provide localized ATP and ROS production at focal adhesion complexes, thereby supporting actin remodeling, focal adhesion turnover, and signaling events that promote the EMT program [12,19].

Here, we demonstrate that SNPH expression is intimately linked to the regulation of the EMT program in SNU-638 human gastric cancer cells. Knockdown of SNPH significantly stimulated the migratory and invasive capacities of SNU-638 cells. This phenotypic shift was accompanied by a characteristic switch in EMT-related gene expression profiles and was driven by the activation of the focal adhesion kinase (FAK)/NF-κB/MMP-9 signaling pathway. Conversely, SNPH overexpression completely reversed these pro-metastatic effects. Collectively, these findings indicate that SNPH functions as a tumor suppressor that abrogates EMT, migration, and invasion in human gastric cancer cells by inhibiting FAK activation.

## 2. Results

### 2.1. Knockdown of Syntaphilin (SNPH) Promotes the Migration and Invasion of SNU-638 Cells

**Figure 1:**
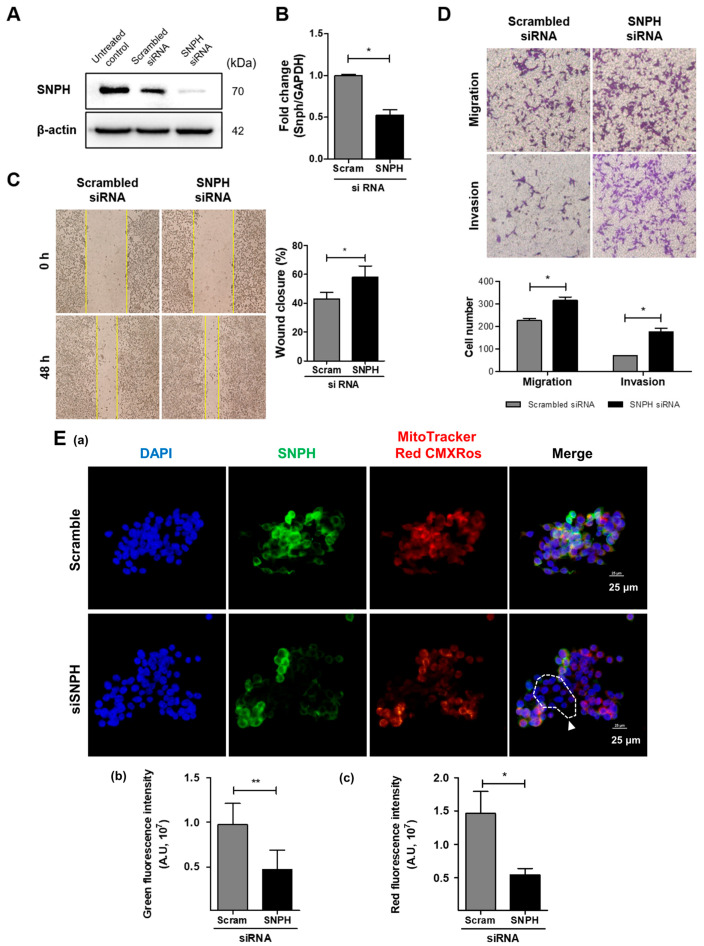
Effects of SNPH knockdown on SNU-638 cell migration and invasion ability. SNU-638 cells were transfected with scrambled siRNA or SNPH siRNA using Lipofectamine RNAiMAX reagent. After 24 h, cells were lysed, and (**A**) SNPH knockdown by SNPH siRNA were verified by Western blot analysis. β-actin was used as an internal control. (**B**) SNPH mRNA expression levels were confirmed by RT-qPCR. (**C**) Wound healing assay was performed to investigate wound-healing ability by SNPH knockdown. The area of the healed wound was measured using Image J v1.53e and shown in a graph. (**D**) Cell migration was performed using transwell. SNU-638 cells were seeded in the upper chamber, and after 24 h, the cells were transfected with scrambled or SNPH siRNA using Lipofectamine RNAiMAX reagent. After 48 h, migrated cells were stained using 2% crystal violet. Cell invasion was measured using transwell coated with matrigel. Likewise, 48 h after transfection, invaded cells were stained using 0.2% crystal violet. Migrated or invaded cells were quantified using Image J. (**E**) Mitochondrial membrane potential was assessed using MitoTracker Red CMXRos: (**a**) fluorescence images, (**b**) green fluorescence intensity, and (**c**) green fluorescence intensity. Red fluorescence indicates active mitochondria, and nuclei were stained with DAPI (blue). Scale bar = 25 μm. All experiments were performed independently three times. Data are presented as mean ± S.D. (*n* = 3, three independent experiments). * *p* < 0.05, ** *p* < 0.01.

To evaluate the impact of SNPH depletion on the motility of SNU-638 gastric cancer cells, we silenced SNPH expression using small interfering RNA (siRNA). Successful knockdown of SNPH at both the mRNA and protein levels was verified via quantitative real-time PCR and Western blotting, respectively (Figure 1A,B). We subsequently performed a scratch wound-healing assay to assess alterations in lateral migratory capacity. Notably, the wound-closure rate was markedly accelerated in the SNPH siRNA-treated group compared to the scrambled control group (Figure 1C). To further investigate whether SNPH deficiency affects the vertical chemotactic migration and invasion of SNU-638 cells, transwell assays were utilized. Consistently, SNPH knockdown significantly enhanced both the migratory and invasive capabilities of SNU-638 cells (Figure 1D), indicating that SNPH loss confers a pro-metastatic phenotype. SNPH knockdown decreased the mitochondrial membrane potential in SNU-638 cells (Figure 1E).

### 2.2. Overexpression of SNPH Suppresses the Migration and Invasion of SNU-638 Cells

**Figure 2:**
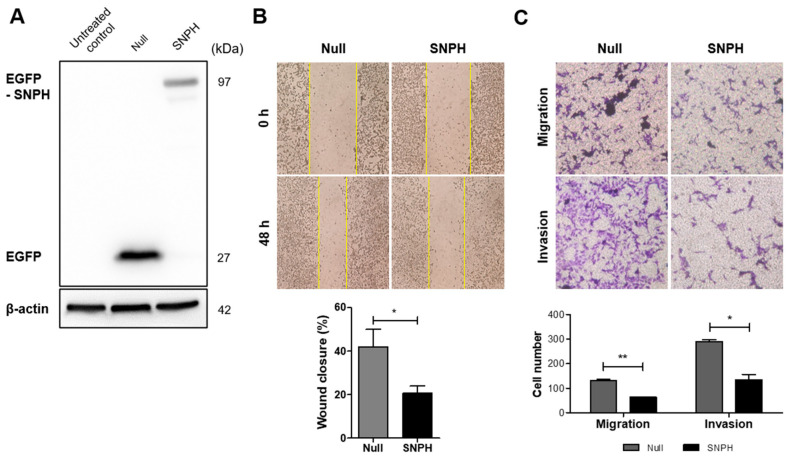
Effects of SNPH overexpression on SNU-638 cell migration and invasion ability. SNU-638 cells were transfected with pcDNA-EGFP-Null or pcDNA-EGFP-SNPH vector using Lipofectamine 2000 reagent. After 24 h, cells were lysed, and (**A**) overexpression of SNPH protein was verified by Western blot analysis. β-actin was used as an internal control. (**B**) Wound healing assay was performed to investigate wound-healing ability by SNPH overexpression. The area of the healed wound was measured using Image J and shown in a graph. (**C**) Cell migration was performed using transwell. SNU-638 cells were seeded in the upper chamber, and after 24 h, the cells were transfected with pcDNA-EGFP-Null or pcDNA-EGFP-SNPH vector using Lipofectamine 2000 reagent. After 48 h, migrated cells were stained using 0.2% crystal. Cell invasion was measured using transwell coated with matrigel. Likewise, 48 h after transfection, invaded cells were stained using 0.2% crystal violet. Migrated or invaded cells were quantified using Image J Data are presented as mean ± S.D. (*n* = 3, three independent experiments). * *p* < 0.05 and ** *p* < 0.01.

Conversely, to determine whether ectopic upregulation of SNPH mitigates the metastatic potential of SNU-638 cells, an SNPH overexpression vector was introduced. Robust overexpression of SNPH in SNU-638 cells was successfully verified via Western blot analysis (Figure 2A). A scratch wound-healing assay demonstrated that the wound-closure rate of SNU-638 cells was significantly retarded following SNPH overexpression (Figure 2B). Furthermore, transwell migration and invasion assays revealed that forced expression of SNPH markedly diminished both the migratory and invasive capacities of the cells (Figure 2C). Taken together, these loss- and gain-of-function experiments consistently demonstrate that SNPH acts as a critical negative regulator of migration and invasion in SNU-638 human gastric cancer cells.

### 2.3. SNPH Regulates EMT Marker Expression and Cytoskeletal Remodeling in SNU-638 Cells

**Figure 3:**
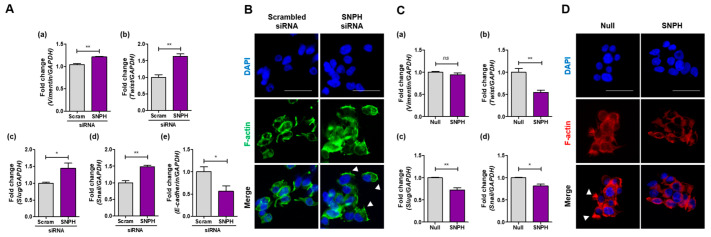
Effects of SNPH on EMT markers and morphological changes in SNU-638 cells. SNU-638 cells were transfected with scrambled siRNA or SNPH siRNA using Lipofectamine RNAiMAX reagent for 24 h. (**A**) RT-qPCR was performed to measure the mRNA levels of (**a**) Vimentin, (**b**) Twist, (**c**) Slug, and (**d**) Snail (**e**) E-cadherin by SNPH knockdown in SNU-638 cells. (**B**) F-actin staining was performed to examine the cell morphology change of SNU-638 cells by SNPH knockdown. Images were observed under a fluorescence microscope. SNU-638 cells were transfected with pcDNA-EGFP-Null or pcDNA-EGFP-SNPH vector using Lipofectamine 2000 reagent for 24 h. (**C**) RT-qPCR was performed to measure the mRNA levels of (**a**) Vimentin, (**b**) Twist, (**c**) Slug, and (**d**) Snail by overexpression of SNPH in SNU-638 cells. (**D**) F-actin staining was performed to examine the cell morphology change of SNU-638 cells by overexpression of SNPH. Images were observed under a fluorescence microscope. White arrowheads indicate membrane protrusions. 200× magnification. Scale bar = 50 μm. Data are presented as the mean ± S.D. (*n* = 3, three independent experiments). * *p* < 0.05 and ** *p* < 0.01.

To elucidate the molecular mechanisms underlying SNPH-mediated regulation of SNU-638 cell motility, we evaluated the transcript levels of key EMT-related markers. Compared with the scrambled control, siRNA-mediated knockdown of SNPH significantly upregulated the mRNA expression of the mesenchymal marker vimentin (VIM), as well as the core EMT transcription factors TWIST, SNAI1 (Snail), and SNAI2 (Slug) (Figure 3A). Notably, TWIST mRNA levels exhibited the most pronounced increase, exceeding 1.5-fold relative to the control (Figure 3A(a)). We concurrently assessed morphological alterations in the cytoskeletal framework by visualizing F-actin using phalloidin staining. SNPH depletion induced the formation of spike-shaped membrane protrusions (indicated by white arrowheads) that are characteristic of highly motile cells (Figure 3B). To further investigate whether SNPH regulates epithelial characteristics during EMT, we measured the transcript levels of the primary epithelial marker, E-cadherin. As shown in Figure 3A(e), siRNA-mediated knockdown of SNPH significantly decreased E-cadherin mRNA expression in SNU-638 cells, confirming the concomitant loss of epithelial adhesive properties alongside the acquisition of mesenchymal features. Conversely, we examined the impact of forced SNPH overexpression on these EMT characteristics. While SNPH overexpression did not alter vimentin mRNA expression (Figure 3C(a)), it significantly downregulated the transcript levels of TWIST, SNAI1, and SNAI2 (Figure 3C(b,d)), with TWIST showing the most significant suppression (Figure 3C(b)). Consistent with these molecular changes, ectopic expression of SNPH effectively abolished the formation of the spike-shaped cytoplasmic protrusions (Figure 3D).

### 2.4. SNPH Regulates FAK Phosphorylation and MMP-9 Expression in SNU-638 Cells

**Figure 4:**
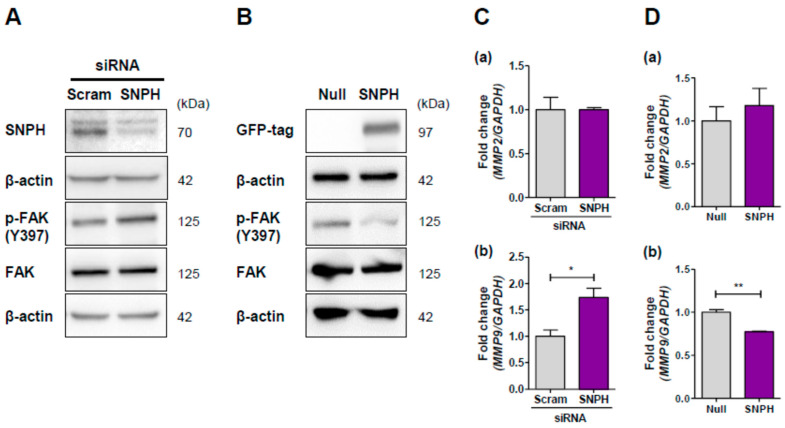
Regulation of MMP-9 expression through activated FAK by SNPH in SNU-638 cells. (**A**) Phosphorylation of FAK by knockdown of SNPH was measured using Western blot assay. Knockdown of SNPH by siRNA was verified (top panel). β-actin was used as an internal control. (**B**) Phosphorylation of FAK by overexpression of SNPH was measured using western blot assay. Overexpression of SNPH was verified. (upper panel, GFP-tagged SNPH). β-actin was used as an internal control. (**C**) RT-qPCR was performed to measure the mRNA levels of (**a**) MMP-2 and (**b**) MMP-9 in cells with SNPH knockdown. (**D**) RT-qPCR was performed to assess the mRNA levels of (**a**) MMP-2 and (**b**) MMP-9 in cells with SNPH overexpression. Data are presented as mean ± S.D. (*n* = 3, three independent experiments). * *p* < 0.05 and ** *p* < 0.01.

Focal adhesion kinase (FAK) is a cytoplasmic protein tyrosine kinase that integrates signaling from integrins and cell surface receptors to promote cell motility and EMT. Downstream of this cascade, FAK signaling is documented to upregulate key EMT markers, including vimentin and Snail. To determine whether SNPH modulates FAK activation, we assessed its phosphorylation status. siRNA-mediated knockdown of SNPH robustly increased FAK phosphorylation (Figure 4A), whereas ectopic overexpression of SNPH significantly diminished FAK phosphorylation (Figure 4B). Given that matrix metalloproteinases (MMPs) are proteolytic enzymes critical for extracellular matrix and basement membrane degradation during tumor invasion, we next investigated whether SNPH selectively influences the expression of the gelatinases MMP-2 and MMP-9. Interestingly, SNPH depletion specifically upregulated MMP-9 mRNA expression without altering MMP-2 levels (Figure 4C). Consistently, forced overexpression of SNPH significantly suppressed MMP-9 transcript levels but exerted no effect on MMP-2 expression (Figure 4D).

### 2.5. Knockdown of SNPH Induces the Phosphorylation and Nuclear Translocation of NF-κB p65 in SNU-638 Cells

**Figure 5:**
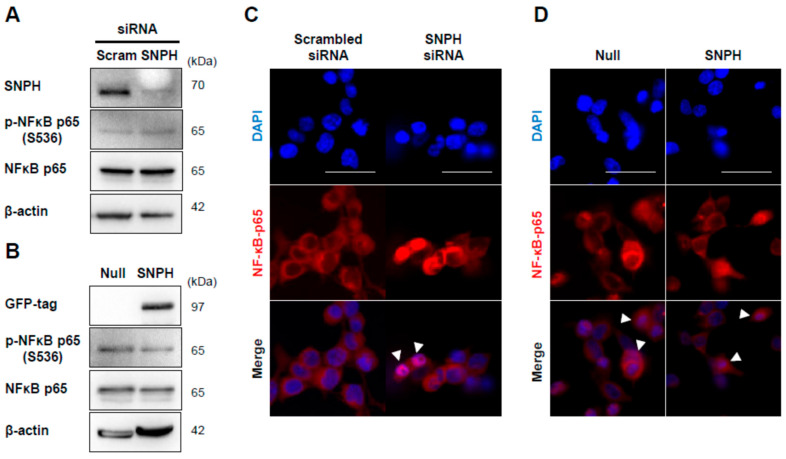
Effects of SNPH on NF-κB activation and translocation in SNU-638 cells (**A**) The activation of nuclear factor-κB (NF-κB) by SNPH knockdown was evaluated through Western blot analysis. Knockdown of SNPH by siRNA was verified (top panel). (**B**) The activation of NF-κB by SNPH overexpression was also assessed through western blot analysis. Overexpression of SNPH was verified (upper panel, GFP-tagged SNPH). The translocation NF-κB p65 to the nucleus was analyzed by immunofluorscence staining. (**C**) SNU-638 cells were transfected with scrambled or SNPH siRNA. After 24 h, they were fixed with 4% formaldehyde and stained using p65 antibody (Red). (**D**) Cells were transfected with pcDNA-EGFP-Null or pcDNA-EGFP-SNPH vector. After 24 h, they were fixed with 4% formaldehyde and stained using p65 antibody (Red). Images were observed under a fluorescence microscope. White arrowheads indicate translocated NF-κB p65. 200× magnification. Scale bar = 50 μm.

Next, we investigated the phosphorylation status of nuclear factor kappa B (NF-κB), a pivotal transcription factor that regulates MMP expression downstream of FAK. siRNA-mediated knockdown of SNPH robustly induced the phosphorylation of NF-κB p65 (Figure 5A), whereas ectopic overexpression of SNPH significantly suppressed its phosphorylation (Figure 5B). Because the nuclear translocation of NF-κB is a prerequisite step for its binding to the promoters of target genes and subsequent transcriptional activation, we examined whether SNPH modulates the subcellular localization of NF-κB p65. Immunofluorescence staining revealed that SNPH knockdown triggered a distinct translocation of NF-κB p65 from the cytoplasm to the nucleus (Figure 5C). Conversely, no obvious alterations in subcellular distribution were observed in SNPH-overexpressing cells (Figure 5D). These results indicate that SNPH deficiency promotes the transcriptional activity of NF-κB by facilitating its phosphorylation and nuclear import.

### 2.6. SNPH Knockdown Promotes EMT and Invasiveness via the FAK/NF-κB/MMP-9 Signaling Pathway in SNU-638 Cells

**Figure 6:**
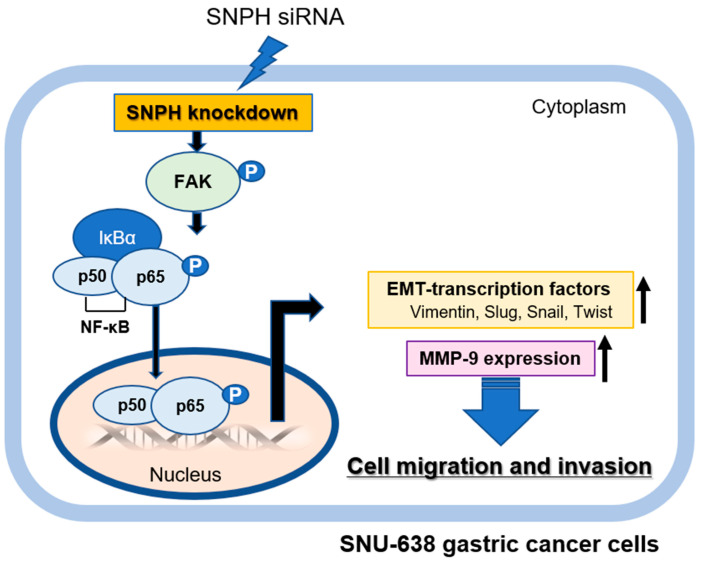
The activation of FAK/NF-κB/MMP-9 signaling pathway by SNPH knockdown in SNU-638 cells. A schematic model illustrating the induction of the EMT process by SNPH knockdown in SNU-638 cells.

Collectively, our findings demonstrate that the knockdown of SNPH induces FAK phosphorylation, which subsequently triggers the activation and nuclear translocation of NF-κB p65. Once in the nucleus, NF-κB drives the transcriptional upregulation of MMP-9. These results compellingly suggest that SNPH deficiency accelerates the EMT program alongside the migratory and invasive capacities of SNU-638 human gastric cancer cells by robustly activating the FAK/NF-κB/MMP-9 signaling cascade (Figure 6).

## 3. Discussion

Although SNPH was initially characterized as a neuron-specific protein, it is now recognized to be widely expressed across various malignancies [12,18]. Mechanistically, SNPH inhibits mitochondrial trafficking to the cortical cytoskeleton, thereby restricting tumor cell migration and metastasis. Furthermore, genome-wide database analyses have revealed that SNPH expression is progressively downregulated or lost during clinical tumor progression, a phenomenon strongly correlated with poor patient outcomes [13,20,21]. Reflecting its functional versatility, emerging studies have underscored the importance of SNPH-mediated mitochondrial redistribution in diverse oncogenic processes, including cell-cycle checkpoint signaling, cancer stem cell (CSC) differentiation, and radioresistance-associated EMT in esophageal squamous cell carcinoma (ESCC) [18,22]. However, despite these insights, the precise regulatory role and downstream mechanisms of SNPH in the specific context of gastric cancer (GC) progression and metastasis have remained entirely unelucidated [18,22]. In the present study, we identified SNPH as a critical suppressor of GC metastasis. While SNPH knockdown promoted EMT and enhanced the metastatic potential of GC cells, its ectopic overexpression completely reversed these pro-metastatic traits. Collectively, our findings suggest that SNPH represents a promising prognostic biomarker and a novel therapeutic target for mitigating gastric cancer progression.

Tumor cell invasion into the surrounding microenvironment is a critical step in cancer progression that enables subsequent metastasis [23,24]. This process requires a phenotypic switch toward a highly migratory state, driven by coordinated alterations in focal adhesion and cytoskeletal dynamics, alongside the upregulation of matrix metalloproteinases (MMPs) to facilitate extracellular matrix (ECM) degradation [25,26]. The epithelial–mesenchymal transition (EMT), orchestrated by a core network of transcription factors, fundamentally supports the acquisition of these invasive traits. EMT not only upregulates MMPs but also reprograms adhesion–cytoskeleton dynamics, in which focal adhesion kinase (FAK) signaling is central [27,28,29]. Structurally, FAK signaling plays a pivotal role in modulating the formation and turnover of focal adhesions. Because FAK activation drives focal adhesion turnover and cell migration, aberrant FAK activation has been widely implicated in the hyper-metastatic behavior and therapeutic resistance of multiple solid tumors [30,31,32]. In this context, our findings provide a novel mechanistic insight by demonstrating that both loss- and gain-of-function manipulations of SNPH bidirectionally and consistently regulate FAK phosphorylation (e.g., pY397). This regulation establishes SNPH as a critical upstream checkpoint controlling the FAK signaling cascade in gastric cancer (GC) cells. Given that SNPH serves as a mitochondrial static anchor, we conceptualize that SNPH-mediated mitochondrial immobilization may spatially restrict localized ATP supply at the cell periphery, thereby limiting the energy-dependent FAK activation and focal adhesion remodeling required for migration and invasion [33,34]. Although we did not directly assess mitochondrial localization, ATP production, ROS generation, or mitochondrial dynamics, our preliminary data showed that SNPH knockdown decreased the mitochondrial membrane potential in SNU-638 cells (Figure 1E). These findings suggest that the loss of SNPH may alter mitochondrial homeostasis, which could subsequently contribute to the enhanced migratory and invasive phenotypes observed in gastric cancer cells. SNPH-mediated regulation of mitochondrial trafficking may mechanistically intersect with FAK signaling by modulating the spatial distribution of mitochondria near focal adhesion sites, thereby allowing localized ATP production and redox signaling to support focal adhesion turnover and FAK activation. In this context, SNPH loss may create a bioenergetic state favorable for enhanced migratory and invasive behavior [13,33,35,36]. Collectively, these insights provide mechanistic rather than purely descriptive evidence and underscore that SNPH modulates GC cell migration and invasion by tightly governing the activation kinetics of the FAK pathway.

The epithelial–mesenchymal transition (EMT) is primarily orchestrated by core transcription factors, including Snail, Slug, and Twist, collectively designated as EMT transcription factors (EMT-TFs) [37,38,39]. In the present study, we evaluated whether syntaphilin (SNPH) modulates the expression of these EMT-TFs alongside the gelatinases MMP-2 and MMP-9 in SNU-638 human gastric cancer (GC) cells. Interestingly, while SNPH expression altered MMP-9 transcript levels, MMP-2 expression remained unchanged. Similarly, loss- or gain-of-function manipulations of SNPH significantly modulated the mRNA expression of key EMT-TFs, such as Snail, Slug, and Twist, with Twist exhibiting the most pronounced transcriptomic fluctuations.

Clinically, the regulatory mechanisms of EMT in GC vary distinctly between intestinal and diffuse histological subtypes. The diffuse type typically features a higher frequency of EMT activation and correlates with a worse prognosis compared to the intestinal type, driven by distinct mechanisms of E-cadherin suppression [40,41]. Intriguingly, Twist serves as the primary driver of EMT in diffuse-type GC, whereas Smad-interacting protein 1 (SIP1) dominates in the intestinal variant [42,43,44]. Consistently, targeted inhibition of Twist in diffuse GC cells has been shown to suppress migration and invasion [43,45,46], while Slug expression correlates strongly with disease progression and distant metastasis across both subtypes [47,48,49]. Given that our molecular data revealed Twist to be the most sensitive downstream target of SNPH, and that SNPH deficiency concurrently induced highly motile cytoskeletal membrane protrusions, our findings suggest that SNPH may function as a critical upstream checkpoint, particularly mitigating the aggressive, Twist-driven diffuse-type GC metastasis. A key finding in our study is that SNPH deficiency simultaneously drives the downregulation of E-cadherin and the upregulation of mesenchymal elements, accompanied by actin cytoskeletal remodeling into spike-shaped protrusions. However, as other markers like N-cadherin and ZEB1/2 were not fully characterized, our current evidence may point toward the induction of a ‘partial EMT’ program. Emerging evidence suggests that a hybrid or partial EMT state confers superior tumor plasticity, anoikis resistance, and enhanced collective cell migration compared to a complete EMT phenotype [50,51,52]. Therefore, the SNPH-mediated partial EMT state might serve as a crucial driver of invasiveness in gastric cancer cells, a molecular landscape that warrants deeper exploration in follow-up studies. EMT is orchestrated by multiple transcriptional regulators, including Snail, Slug, Twist, and the ZEB family [53,54,55]. In particular, ZEB1 and ZEB2 have been implicated in sustaining mesenchymal traits, suppressing epithelial characteristics, and promoting invasion and metastasis in various cancers [56]. Although the present study did not assess ZEB1/2 directly, our findings that SNPH depletion increases mesenchymal markers and EMT-associated transcription factors, together with reduced E-cadherin expression, are consistent with a broader EMT program in which ZEB family members may also be involved [54,57]. This possibility warrants further investigation.

Furthermore, we confirmed that SNPH regulates the activation kinetics of nuclear factor kappa B (NF-κB), a key transcriptional regulator of MMP-9 expression [58]. While SNPH knockdown triggered the phosphorylation and subsequent nuclear translocation of the NF-κB p65 subunit, ectopic SNPH overexpression effectively blocked this nuclear import compared to null control cells. Collectively, these data indicate that the SNPH/FAK axis finely tunes GC metastatic potential by co-regulating EMT-TFs and NF-κB-mediated ECM degradation, providing a rational basis for targeting SNPH in advanced gastric malignancies.

Although our bi-directional loss- and gain-of-function experiments strongly point to the activation of the FAK/NF-κB/MMP-9 cascade upon SNPH depletion, a limitation of the current study is the lack of direct rescue or inhibition experiments utilizing specific pharmacological inhibitors or siRNAs targeting FAK, NF-κB, or MMP-9. Further investigations incorporating these functional blocking or rescue strategies will be essential to definitively confirm the dependency of SNPH-depleted gastric cancer cell motility on this signaling pathway. In this study, SNU-638 cells were strategically selected as our experimental model based on pre-screening evaluations of baseline migration dynamics among five gastric cancer cell lines (Supplementary Figure S1). SNU-638 represented an optimal biological window with moderate migration kinetics suitable for bidirectional functional assays. Nevertheless, given the high heterogeneity of gastric cancer, the reliance on a single cell line is a limitation of this study. Further validation across different gastric cancer cell lines with diverse molecular backgrounds is warranted and is currently ongoing as part of our follow-up investigation. Lastly, it is important to note that the findings of the present study rely exclusively on in vitro functional assays. While our loss- and gain-of-function data strongly support the regulatory role of SNPH in cell migration, invasion, and EMT, the lack of in vivo validation using xenograft or orthotopic gastric cancer metastasis models remains a limitation. Given that the in vivo tumor microenvironment incorporates complex systemic factors, further preclinical animal studies will be essential to definitively confirm the translational potential of targeting the SNPH axis to suppress gastric cancer metastasis. It must be acknowledged, however, that the potential clinical utility of SNPH as a prognostic indicator or therapeutic vulnerability remains speculative at this stage, as our study did not incorporate direct analyses of clinical samples or patient-derived datasets. Although public genome-wide databases suggest that down-regulated SNPH expression correlates with advanced clinical stages and poor survival in multiple malignancies, further rigorous translational studies utilizing large cohorts of human gastric cancer tissue specimens will be indispensable to definitively validate the clinical and diagnostic feasibility of targeting the SNPH/FAK axis [12,14,22].

In conclusion, our findings demonstrate that SNPH deficiency drives the EMT program and cellular invasiveness via the FAK/NF-κB/MMP-9 signaling axis in SNU-638 human gastric cancer cells. In addition to the SNPH/FAK/NF-κB/MMP-9 axis described here, other EMT-related signaling pathways, including TGF-β, Wnt, Notch, Hedgehog, PI3K/AKT, and MAPK/ERK, may also contribute to SNPH-mediated phenotypic regulation [59]. Future studies will be required to determine whether SNPH intersects with these pathways in gastric cancer progression. Furthermore, we propose that SNPH functions as a pivotal molecular rheostat governing the epithelial–mesenchymal transition and metastatic progression of gastric cancer, highlighting its potential as a novel prognostic indicator and an attractive therapeutic vulnerability.

## 4. Materials and Methods

### 4.1. Cell Culture

Human gastric cancer cell line SNU-638 was obtained from the Korea cell line Bank (KCLB, Seoul, Republic of Korea). SNU-638 cells cultured in RPMI-1640 medium (Welgene, Gyeongsan, Republic of Korea) containing 10% fetal bovine serum (FBS, Welgene) and 1% penicillin/streptomycin (Welgene). The cells were incubated in a humidified atmosphere of 5% CO_2_ at 37 °C.

### 4.2. Plasmid DNA Vector and Small Interfering RNA (siRNA) Transfection

SNPH was amplified using PCR and then cloned into the pcDNA3-EGFP plasmid between EcoRI/XhoI sites to construct the SNPH expression vector. pcDNA3-EGFP plasmid was purchased from Addgene (Watertown, MA, USA). SNU-638 cells were transfected with pcDNA3-EGFP-Null or pcDNA3-EGFP-SNPH vector using Lipofectamine^TM^ 2000 transfection reagent (Invitrogen, Carlsbad, CA, USA) according to the manufacturer’s instructions. And, for knockdown of SNPH, cells were transfected with control siRNA or SNPH siRNA (SantaCruz, Starr County, TX, USA) using Lipofectamine RNAiMAX transfection reagent (Invitrogen) according to the manufacturer’s instructions.

### 4.3. Wound Healing Assay

SNU-638 cells were seeded in 12-well plates at 3 × 10^5^ cells/mL and grown in an incubator for 1 day to reach 70–80% confluency. And then, the cells were transfected with siRNA or plasmid DNA constructs. After 24 h, wounds were scratched onto the cell monolayer with a yellow tip. The cells were then cultured at 37 °C with 5% CO_2_ and images were acquired at 0, 24, and 48 h. Wound closure area (%) was determined using Image J software.

### 4.4. Transwell Migration and Invasion Assay

Cell migration and invasion abilities were confirmed using 6.5 mm transwell polycarbonate membrane cell culture inserts with 8.0 μm pore size (Corning, Corning, NY, USA). For migration assay, 2 × 10^4^ cells were placed in the upper chamber of the insert. For invasion assay, the transwell insert was coated with matrigel (BD Bioscience, Franklin Lakes, NJ, USA) and 2 × 10^4^ cells were plated on the upper part of the coated filter. The medium 600 μL containing 10% fetal bovine serum was placed in the lower chamber. siRNA or plasmid DNA was transfected 24 h later. After 48 h of incubation, cells remaining on the top of the transwell inserts were removed with a cotton swab, and the insert was stained with 0.2% crystal violet and observed under a microscope.

### 4.5. Quantification of Fluorescence Intensity

Fluorescence intensity was quantified using ImageJ software v1.53e. Fluorescence images were acquired under identical acquisition settings, including exposure time, laser intensity, and detector gain, for all experimental groups. For quantitative analysis, the green fluorescence channel representing SNPH and the red fluorescence channel representing mitochondria (MitoTracker Red CMXRos, Thermo Fisher Scientific, Waltham, MA, USA) were analyzed separately. The mean fluorescence intensity (Mean Gray Value) of the image was measured using the Measure function in ImageJ. The quantified fluorescence intensity bar graph was presented using GraphPad Prism software.

### 4.6. Immunofluorescence Assay

SNU-638 cells were seeded in 12-well plates at 1 × 10^5^ cells/mL on the coverslips. After 24 h, the cells were transfected with siRNA or plasmid DNA constructs. The cells were fixed with 4% paraformaldehyde (PFA) for 10 min and then washed three times for 5 min each with PBS. Subsequently, the cells were blocked with blocking buffer (5% BSA, 0.3% Triton X-100 in PBS) for 1 h at room temperature. And incubated overnight at 4 °C with primary antibodies. After being washed with PBS, followed by incubation with rhodamine red X or FITC-conjugated IgG secondary antibody for 1 h. The cells were mounted with Vectashield (Vecta Laboratories, Inc., Dallas, TX, USA) containing DAPI and analyzed under a fluorescence microscope (Olympus).

### 4.7. F-Actin Staining

SNU-638 cells were seeded in 12-well plates at 1 × 10^5^ cells/mL on the coverslips. The cells were fixed with 4% paraformaldehyde (PFA) for 10 min and then washed three times for 5 min each with PBS. Subsequently, the cells were blocked with blocking buffer (5% BSA, 0.3% Triton X-100 in PBS) for 1 h at room temperature, and three washed with PBS. The cells were added to the Alexa Fluor 488 or DyLight 594 phalloidin solution (Cell Signaling Technology, Inc., Danvers, MA, USA) and incubated for 15 min, followed by three washed with PBS. The cells were mounted with Vectashield (Vecta Laboratories, Inc., Dallas, TX, USA) containing DAPI and analyzed under a fluorescence microscope (Olympus, Tokyo, Japan).

### 4.8. Reverse Transcription-PCR (RT-PCR) and Quantitative Real-Time PCR (qPCR)

Total RNA was extracted using easy-BLUE Total Extraction Kit (iNtRON, Seongnam, Republic of Korea) according to the manufacturer’s instruction. Then, cDNA was synthesized using PrimeScript RT Reagent Kit (Takara, Shiga, Japan) according to the manufacturer’s instructions. Real-time quantitative PCR was performed using Power SYBR Green PCR Master Mix (Applied Biosystems, Warrington, UK), and the device used was QuantStudio^TM^ 3 Real Time PCR System (Applied Biosystems, Foster City, CA, USA). After all cycles were completed, melting curve analysis was performed to confirm the specificity of the primer. The results were analyzed using Real-Time PCR Instrument software (v1.5.1) provided by Applied Biosystems. The primer sequences used for PCR are listed in Table 1.

### 4.9. Western Blot Analysis

SNU-638 cells were lysed in RIPA buffer (Sigma-Aldrich, St. Louis, MO, USA) containing protease and phosphatase inhibitors (Sigma-Aldrich). Protein concentrations were determined using the BCA assay (iNtRON, Seongnam, Republic of Korea). Proteins (30 μg) were separated by SDS-PAGE gel, and transferred to PVDF membrane (Millipore, Burlington, MA, USA). The membranes were blocked with 5% skim milk dissolved in TBST buffer. And incubated overnight at 4 °C with primary antibodies and incubated with HRP-conjugated secondary antibodies (Cell Signaling Technology) for 1 h at room temperature. β-actin (Sigma-Aldrich) was used as a loading control. Then, the membrane was detected using SuperSignal West Pico Chemiluminescent substrate (Pierce, Rockford, IL, USA). Images of the blots were visualized by ChemiDoc XRS + Imaging system (BioRad, Hercules, CA, USA). Densitometric values for each band were measured using the Image J software (National Institutes of Health, Bethesda, MD, USA).

### 4.10. Statistical Analysis

The data were analyzed using GraphPad Prism 5 and represented as mean ± S.D (standard deviation). Comparisons between the data from two groups were performed using Student’s *t*-test. *p* < 0.05 was considered to be statistically significant.

## Figures and Tables

**Table 1:** Primer sequences for RT-qPCR.

Gene (5′ -> 3′)		Primer Sequences
*hSNPH*	Forward	5′-TCAGGGTTGTTGAGAGGAGTCA-3′
	Reverse	5′-CCAGTTGGCCCGTGGTT-3′
*hMMP2*	Forward	5′-CAAGGACCGGTTCATTTGGC-3′
	Reverse	5′-ATTCCCTGCAAAGAACACAGC-3′
*hMMP9*	Forward	5′-TTGACAGCGACAAGAAGTGG-3′
	Reverse	5′-GCCATTCACGTCGTCCTTAT-3′
*hVIMENTIN*	Forward	5′-CAGGAGGCAGAAGAATGGTAC-3′
	Reverse	5′-TTAAGGGCATCCACTTCACAG-3′
*hTWIST*	Forward	5′-CTACGCCTTCTCGGTCTG-3′
	Reverse	5′-CTTCTCTGGAAACAATGACATCT-3′
*hSLUG*	Forward	5′-CATCTTTGGGGCGAGTGAGT-3′
	Reverse	5′-ATGGCATGGGGGTCTGAAAG-3′
*hSNAIL*	Forward	5′-GTTTACCTTCCAGCAGCCCT-3′
	Reverse	5′-TCCCAGATGAGCATTGGCAG-3′
*hGAPDH*	Forward	5′-GGCATGGACTGTGGTCATGAG-3′
	Reverse	5′-TGCACCACCAACTGCTTAGC-3′

## Data Availability

The original contributions presented in this study are included in the article/Supplementary Materials. Further inquiries can be directed to the corresponding author(s).

## Supplementary Materials

The following supporting information can be downloaded at https://www.mdpi.com/article/10.3390/ijms27156564/s1.
