# Supplementary material for: Syntaphilin Regulates Epithelial-Mesenchymal Transition and Metastasis in Gastric Cancer via the FAK/NF-κB/MMP-9 Signaling Pathway
Source: Int J Mol Sci. 2026 Jul 23;27(15):6564. doi: 10.3390/ijms27156564 (PMC13465459; doi:10.3390/ijms27156564)
Supplement: Supplementary file 1 [file ijms-27-06564-s001.zip › ijms-4403943-supplementary.pdf]

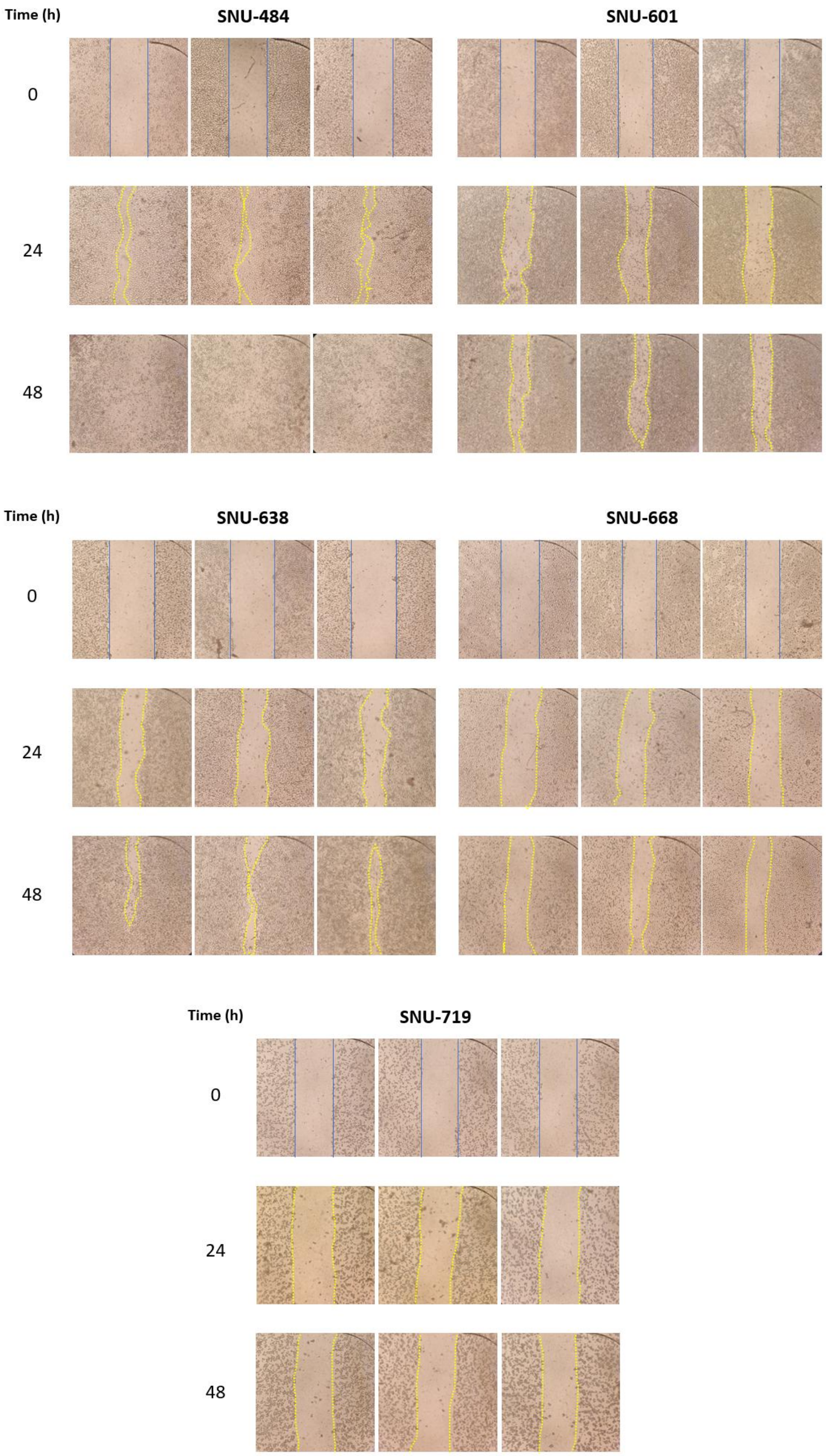

Supplementary Figure S1. Baseline scratch wound-healing capacities of five human gastric cancer cell lines (SNU-484, SNU-601, SNU-638, SNU-668, and SNU-719) at 0, 24, and 48 hours.
